# Ultra-Processed Food Intake is Associated with Altered Glucose Homeostasis in Young Adults

**DOI:** 10.21203/rs.3.rs-6875960/v1

**Published:** 2025-07-01

**Authors:** Yiping Li, Elizabeth Costello, Sarah Rock, William B. Patterson, Zhanghua Chen, Frank Gilliland, Michael I. Goran, Tanya L. Alderete, Jesse A. Goodrich, David V. Conti, Nikos Stratakis, Leda Chatzi

**Affiliations:** University of Southern California; University of Southern California; University of Southern California; University of Colorado School of Medicine; University of Southern California; University of Southern California; University of Southern California; Bloomberg School of Public Health, Johns Hopkins University; University of Southern California; University of Southern California; Barcelona Institute for Global Health (ISGlobal); University of Southern California

**Keywords:** ultra-processed food, type 2 diabetes, prediabetes, young adults, body composition

## Abstract

**Background:**

Ultra-processed foods (UPFs), often high in sodium, sugar, and unhealthy fats, compose more than half of total dietary energy consumption in the United States. A diet composed of a high amount of UPFs can contribute to glucose dysregulation and insulin resistance, which may lead to prediabetes and type 2 diabetes (T2D). The goal of this study is to examine associations between UPF consumption and prediabetes and related biomarkers in youth.

**Methods:**

Young adults (n = 85) aged 17–22 years old from the Meta-AIR study, a subset of the Children’s Health Study, were enrolled between 2014–2018 and returned for a second visit between 2020–2022. Participants completed two 24-hour dietary recalls and an oral glucose tolerance test at each visit. Food items were categorized as either an UPF or non-UPF according to NOVA classification guidelines. The proportion of the diet composed of UPFs was calculated for each participant. Regression models were used to assess relationships of UPF consumption at baseline and change between visits with markers of glucose homeostasis at follow-up, adjusting for demographics and physical activity.

**Results:**

A 10 percentage-point increase in UPF consumption between visits was associated with a 64% (OR: 1.64, 95% Cl: 1.15, 2.50) higher risk for prediabetes and 56% (OR: 1.56, 95% CI: 1.42, 5.86) higher risk for impaired glucose tolerance at follow-up. Higher baseline UPF consumption was significantly positively associated with fasting insulin (*β* = 2.09, 95% CI: 0.06, 4.12), 2-hour insulin (*β* = 44.75, 95% CI: 22.26, 67.25) and insulin area under the curve (*β* = 63.19, 95% CI: 34.84, 91.54) at follow-up.

**Conclusion:**

UPF consumption may increase the risk for T2D among young adults. Our findings suggest that limiting UPF consumption could be an important strategy for T2D prevention in this population.

## Introduction

Prediabetes has become more common among young adults in recent years, which increases the risk for early onset type 2 diabetes (T2D)^1,2^. In the United States (US), the prevalence of diagnosed T2D is estimated to be about 48 per 100,000 in youth under 20 years of age^3^. T2D is also a significant global public health concern because it can affect individuals’ quality of life, lead to many comorbidities, and increase mortality risk^2,4^. The early onset of T2D among young adults can lead to more long-term health issues compared to onset of T2D in later adulthood^1,5–9^. Obesity greatly increases the risk for prediabetes and T2D, and poor diet and other lifestyle factors can be risk factors for all three conditions^2,4,5^. Since prediabetes, T2D, and obesity are closely related to each other and share similar risk factors, the assessment of modifiable risk factors like diet is crucial for prevention and treatment of these conditions.

In the US, more than half of total dietary energy consumption is composed of ultra-processed foods (UPFs)^10,11^. UPFs are food items that go through multiple industrial processes before people purchase or eat them^12^. Examples of common UPFs include soft drinks, packaged snacks, margarine, and sausages^12^. Most UPFs are calorie-dense and high in sugar, salt, and unhealthy fats, while low in protein, vitamins, and minerals^10,13–16^. Studies have demonstrated that higher consumption of UPFs results in poor nutritional diet quality and increased risk for the development of chronic diseases, including T2D and hypertension^13–18^. It is important to limit UPF consumption in childhood and adolescence due to the high content of added sugar and saturated fats in UPFs and their possible contribution to weight gain, T2D, cardiovascular disease, and hypertension^16–18^.

Though many risk factors for metabolic disease first appear in early life, most research studies have focused on the effects of UPFs on metabolic disease in middle-aged and older adults^19–22^. Many of these studies show that diets with a higher proportion of UPFs or increasing consumption of UPFs is associated with a higher risk of T2D and obesity among adults^19–22^. In addition, most previous studies examining UPF consumption and metabolic disease have been conducted in Brazil and primarily used cross-sectional analyses, with only a few using a longitudinal study design^23–27^. However, few studies have assessed the associations between UPFs and T2D or obesity in young people, and those that have are cross-sectional and report mixed results^18,20–22^. Some studies have shown that limiting the consumption of UPFs can reduce T2D and obesity risk among children and young adults, while others found no association between UPF consumption and obesity or overweight^18,20–22^. Because of the limited studies on UPF consumption among young adults, and the importance of early lifestyle changes in preventing T2D among high risk populations, more research is needed to understand the relationship between UPF consumption and risk for T2D and obesity in young adults^28^.

The purpose of this study is to assess the longitudinal associations between UPF consumption and prediabetes and obesity in young adults, using glucose and insulin measurements, body composition, and diet assessment over four years of follow-up. We hypothesized that increases in UPF consumption would be associated with a higher risk of altered glucose homeostasis, insulin resistance, obesity, and prediabetes.

## Methods

### Cohort

**Figure 1:**
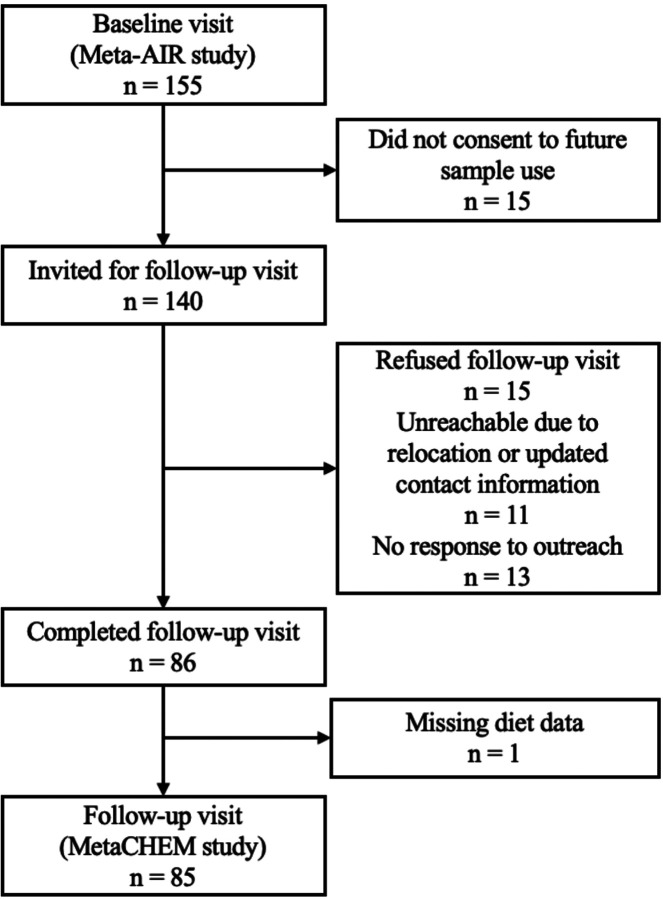
Flowchart for participants recruitment.

Between 2014–2018, 155 young adults aged 17–22 who had previously participated in the Children’s Health Study were invited to enroll in the Meta-AIR study^9^. To be eligible, participants had a history of overweight or obesity in early adolescence, were not diagnosed with either type 1 or type 2 diabetes, were not taking medications that influence glucose metabolism, and had no other significant medical diagnosis^9^. Of these, 85 returned for a follow-up visit between 2020–2022 (the MetaCHEM study, Fig. 1)^9^. This study was approved by the Institutional Review Board at the University of Southern California and written informed consent or assent were obtained from participants and their guardians.

### Dietary Assessment and UPF Classification

Participants completed two non-consecutive 24-hour dietary recalls on one weekday and one weekend day at each visit^9^. Trained interviewers used the Nutritional Data System for Research (NDSR) software version 2014 to complete the baseline recalls, while participants used the Automated Self-Administered 24-hr Dietary Assessment Tool (ASA24) version 2018 to conduct the recalls at the follow-up visit^9,29,30^. At baseline, 10.3% (n = 16) of participants completed only one recall, while at follow up 10.2% (n = 9) of participant completed only one recall.

In this study, a total of 1,167 unique food items were reported at the baseline visit and a total of 807 unique food items were reported the at the follow-up visit. Some food items reported at the follow-up visit contained mixed dishes with multiple ingredients. When possible, these foods were disaggregated into individual ingredients using the 2017–2018 Food and Nutrient Database for Dietary Studies (FNDDS) Ingredients database and matched to the food codes from the FNDDS Foods and Beverages database that were provided by ASA24^9,31^. Each ingredient or food was classified as ultra-processed or not by two independent reviewers (YL, EC). Foods were classified according to the NOVA group definitions of 1) unprocessed and minimally processed foods, 2) processed culinary ingredients, 3) processed foods, and 4) ultra-processed foods (UPFs)^10,12,32^. Any disagreements on UPF classification were resolved through discussion.

Briefly, unprocessed and minimally processed foods in NOVA group 1 are edible parts of plants or animals and foods that have been through basic processing such as drying, griding and freezing (e.g., fruits and vegetables, eggs, meat and seafood, flour, spices)^12^. Processed culinary ingredients in NOVA group 2 mainly composed of vegetable oils, butter, salt, sugar, and honey for the main purpose of seasoning and cooking^12^. Processed foods in NOVA group 3 contain food products that are made by adding items in group 2 to group 1 for the purpose of preservation (e.g., canned vegetables, cured or smoked meat and fish, freshly made bread)^12^. UPFs in NOVA group 4 are made with series of industrial techniques that cannot be replicated at home and may involve molding or pre-frying, the addition of colors and flavors, and often includes added sugars, salt, oils, and fats^12^. This group also includes most branded and packaged foods, pre-prepared ready-to-eat products, and instant foods^12^. However, enriched food items, like milk or flour with added vitamins or minerals, are not included in this group. Examples of UPFs are soft drinks, candies, cereal, ice-cream, mass-produced cookies or pastries, margarines, packaged and shelf-stable spreads, milk drinks, flavored yogurts, pizza, and sausages.

UPF classification was made with the following considerations: how the food is typically prepared in the U.S. and how most people obtain the food (if purchased from a store, restaurant, or homemade). Items from fast food restaurants were categorized as UPFs. Bread and rolls were presumed to be shelf-stable and purchased from grocery stores or wholesalers were classified as ultra-processed unless reported to be homemade.

### UPF Percentage Calculation

The proportion of the diet composed of UPFs was calculated by weight rather than calories, to account for some foods that provide no contribution to energy intake, such as diet soda. The percent of diet that was ultra-processed (UPF%) was calculated as the total amount of UPFs in grams divided by the total amount of foods and beverages consumed in grams, multiplied by 100% and averaged across both recall days. If a participant only completed one dietary recall, the dietary information from the single day was used. The change in UPF consumption between visits (UPF% *Δ*) was calculated as UPF%_baseline_ – UPF%_follow–up_.

### Study Outcomes

Glucose homeostasis was assessed using hemoglobin A1c (HbA1c) and a 2-hour oral glucose tolerance test (OGTT) glucose and insulin related measures^9^. During the OGTT, glucose and insulin were measured in plasma while fasting and at 30-, 60-, 90- and 120 minutes after glucose administration^9,33^. HbA1c was measured in fasting whole blood samples^9^. The glucose and insulin area under the curves (AUCs) were calculated using the trapezoidal method with the 5 time points from the OGTT^9,33,34^.

Prediabetes or T2D was categorized according to the American Diabetes Association criteria. Participants were considered to have prediabetes if their HbA1c was between 5.7% and 6.4%, their fasting glucose was between 100 mg/dL and 125 mg/dL, or their 2-hour glucose was between 140 mg/dL and 199 mg/dL. Participants were considered to have T2D if their HbA1c was 6.5% or higher, their fasting glucose was 126 mg/dL or higher, or their 2-hour glucose was 200 mg/dL or higher^33,35^. Impaired fasting glucose (IFG) was defined as having a fasting glucose value greater than 100 mg/dL and impaired glucose tolerance (IGT) was defined as having a 2-hour glucose value greater than 140 mg/dL^36^.

To assess insulin resistance and beta-cell function, the homeostatic model assessment of insulin resistance (HOMA-IR) and homeostatic model assessment of β-cell function (HOMA-β) were calculated from fasting glucose and fasting insulin values^37^. The Matsuda Index was used to estimate the insulin sensitivity of the entire body using the 5 time points from the OGTT^33,38^.

Body composition was assessed using body mass index (BMI, kg/m^2^) and dual-energy X-ray absorptiometry (DEXA)^9,39^. DEXA measures included body fat percentage, android to gynoid ratio, fat mass to height ratio (kg/m^2^), and visceral adipose tissue (VAT) mass (g)^9,39^. BMI was categorized as normal weight (< 25 kg/m^2^), overweight (25–29.9 kg/m^2^), and obesity ≥ 30 kg/m^2^) using body weight and height measured at each visit.

### Covariates

Demographic information including age, sex, ethnicity, and physical activity were self-reported though questionnaires at baseline and follow up^9^. Ethnicity was categorized as White, Hispanic/Latino, and Other. Exercise level at the follow-up visit was assessed using the International Physical Activity Questionnaire and categorized into High, Medium, and Low^9,40^.

### Statistical Analysis

Descriptive statistics for %UPF consumption, outcomes, and covariates at both visits were calculated. Differences between categorical variables at each visit were assessed using McNamar’s test and differences between continuous variables at each visit were assessed using paired t-tests. Few participants were found to have T2D, so prediabetes and T2D were combined into one outcome group (Prediabetes/T2D) for analysis.

Linear and logistic regressions were used to evaluate the effects of UPF consumption on each outcome, measured at the follow-up visit. Each model contained UPF% consumption at baseline, UPF% *Δ*, and adjusted for covariates as follows: Δ

$$Y_{\text{outcome}\;\text{variables}}=\beta_{0}+\beta_{\text{UPF}\%\Delta }X_{\text{UPF}\%\Delta }+\beta_{\text{baseline}\;\text{UPF}\%}X_{\text{baseline}\;\text{UPF}\%}+\text{covariates}.$$

All models adjusted for age, sex, race and ethnicity, and exercise at the follow-up visit. Beta estimates and odds ratios were scaled by 10 units. All analyses were performed using R (version 2022.02.3 + 492; R Core Development Team).

## Results

### Descriptive Statistics

Descriptive statistics for participants’ characteristics are presented in Table 1. The proportion of the diet composed of UPFs increased, on average, from about 20% at baseline to almost 24% at follow up (p = 0.02). BMI, T2D, and IGT were not significantly different between visits, while more participants had IFG at the follow-up than at baseline (Table 1). Fasting glucose increased by 4.91mg/dL from the baseline to the follow-up visit (p < 0.05), while HbA1c, two-hour glucose, and glucose AUC also increased between visits, though the increase was not statistically significant (Table 2). Similar patterns between visits were observed where fasting insulin, two-hour insulin, HOMA-*β*, and HOMA-IR increased between visits, though Matsuda index significantly decreased (p < 0.05) (Table 2). All body composition measurements significantly increased from the baseline to the follow-up visit, except android/gynoid ratio (Table 2).

### Prediabetes/T2D and Insulin Resistance

Table 3 shows the associations between UPF% *Δ* and UPF% at baseline and prediabetes/T2D, IFG, and IGT after adjusting for covariates. A 10-unit increase in UPF% *Δ* was significantly associated with 64% higher risk of having prediabetes/T2D (OR: 1.64, 95% CI: 1.15, 2.50), and with a 56% higher risk of having IGT (OR: 1.56, 95% CI: 1.42, 5.86). UPF% *Δ* was also significantly positively associated with 2-hour glucose (β = 5.72, 95% CI: 0.43–11.01) (Table 4). Significant positive associations were also observed between baseline UPF% and fasting insulin, 2-hour insulin, and insulin AUC, and a negative association was observed between baseline UPF% and Matsuda index (Table 4).

### Body Composition

The associations between UPF% *Δ* and UPF% at baseline and body composition measurements are shown in Table 4. There were no statistically significant associations between UPF% *Δ* or baseline UPF% and body composition. However, we observed positive but non-statistically significant associations between UPF% *Δ* and baseline UPF consumption and BMI, body fat percent, android/gynoid ratio, fat mass/height^2^ and VAT mass.

## Discussion

In this novel longitudinal analysis, we found that increasing UPF consumption over a four-year period increased the risk for prediabetes and IGT in young adults. Higher UPF consumption was associated with significant increases in some markers of insulin sensitivity including fasting insulin, 2-hour insulin, and insulin AUC. Positive but non-significant associations with most measures of adiposity were also observed. Increases in UPF consumption between study visits was also associated with decreasing Matsuda index, a measure of insulin sensitivity that describes insulin secretion relative to blood glucose^38^. These findings suggest that UPF consumption is associated with increased risk for insulin resistance. Importantly, since early prevention and T2D treatment among young adults can be highly effective, our results highlight the adverse impact of UPF consumption on T2D development and emphasize the importance on dietary habits for young adults^28^.

Previous studies have investigated associations between UPF consumption and T2D, though none have included detailed glucose and insulin measurements to explore possible mechanisms of T2D development or the changes in glucose homeostasis that could lead to T2D^27,41,42^. In addition to positive associations between UPF consumption and IGT and prediabetes, we also found associations between UPF consumption and insulin resistance by multiple measures: lower Matsuda indices, higher insulin concentrations across the OGTT, and positive, but non-significant, association with HOMA-IR. Insulin resistance and beta-cell dysfunction are important physiological characteristics of T2D and can be influenced by diet. Some nutritional components of many UPFs, including saturated fat, free fatty acids, and added sugars, are known to affect beta-cell function. If consumed in excess, these nutrients could exhaust beta-cells, inhibiting their function and further contributing to insulin resistance and eventually T2D^43^. Though we did not observe any inverse associations between UPF consumption and HOMA-β, this does not exclude beta-cell exhaustion as a possible mechanism underlying the relationship between UPF consumption and T2D; relationships between HOMA-β and risk for T2D are inconsistent across different populations and among populations at different stages of T2D progression^44,45^. It is important to continue to explore the impact of UPF consumption and its underlying role on developing T2D.

Growing proportions of the diet are composed of UPFs, which are usually high in added sugars, saturated fats, or other nutrients, leading to lower diet quality and increasing prevalence of diet-associated chronic diseases^15–18^. Existing studies on UPF consumption have primarily focused on middle-aged or older adults, yet the early onset of T2D among young adults is rising, suggesting the need for evaluation and interventions targeted at youth^1^. Studies in older adults have showed that higher UPF consumption is associated with an increased risk for T2D, which is consistent with our findings^46–50^.

However, evidence for associations between overweight or obesity, both risk factors for T2D, and UPF consumption is inconsistent across different age groups. While many studies have reported a positive association between UPF consumption and overweight and/or obesity among adults, others did not observe the same pattern in children^21,22,41,50,51^. We observed mostly positive, but non-significant, relationships between UPF consumption and obesity-related body compositive measurements, which is consistent with previous studies in adults^23–25^. These studies suggested that reducing UPF consumption may also benefit adults by preventing excess weight gain^23–25^. However, the lack of statistical significance in our findings may be due to the limited sample size, suggesting larger sample sizes are needed for future studies of the impact of UPF consumption on young adults.

Many UPFs are high in salt, fat, and sugar, which could independently contribute to metabolic diseases, and thus are especially relevant targets of public health interventions^52–54^. High salt intake may be a contributor to obesity and diabetes^55–57^, and dietary fat, especially trans fatty acids and saturated fats, is positively associated with T2D and obesity risks due to its effects on insulin sensitivity^57–60^. Consumption of soft drinks and foods that contain high amounts of added sugar were also found to increase risk for T2D and obesity, and limiting added sugar intake and UPFs may prevent chronic disease in children and adolescents^16,61,62^. Previous work and the findings from our present study suggest that the nutrients common to UPFs increase the risk for obesity, which may be a mechanism by which UPFs increase the risk for T2D.

This study has many strengths. Firstly, gold-standard outcome measurements were obtained using OGTT and DEXA at both time points^9^. Secondly, our study focuses on young adults: an age group not often included in previous work. Young adults in their late teens and twenties have only recently reached physically mature and are undergoing significant lifestyle changes that may affect their risk for obesity and T2D. Thirdly, this study is one of the few longitudinal studies to examine the relationship between UPFs and risk for prediabetes, insulin resistance, and obesity, where diet and each outcome was assessed at each time point. This study design allowed us to evaluate the changes in UPF consumption over several years to follow up and assess the resulting impacts on glucose homeostasis and insulin sensitivity. However, this study also has some limitations. Our relatively small sample size may have limited our statistical power to detect associations between UPF consumption and some outcomes. Despite this, we did have enough power to consistently detect associations between UPF% consumption and prediabetes, IGT, and markers of insulin resistance. Additionally, dietary recalls may be subject to recall bias and may not represent long-term eating habits, though administration of multiple recalls improves estimates of nutrient intake and the variety of foods consumed^9^. We also used two different dietary recall systems, NDSR and ASA24, for the baseline and follow-up visits, which use different databases for reported foods. However, both systems provided a similar level of detail about food processing and sources, and we do not expect that one or the other would systematically encourage classification into higher NOVA processing categories^62,63^. While it is possible that some misclassification of UPFs occurred, though we minimized this by preforming the classification using two independent researchers, and we would expect any misclassification to be non-differential.

Findings from this study suggest that reducing UPF consumption may reduce the risk for prediabetes and T2D in youth. Young adults may also benefit from limiting foods that contain high amounts of salt, fat, and sugar as they all potentially contribute to obesity, which also increases the risk for T2D^4,5^. Metabolic diseases such as T2D and obesity are significant public health problems and are becoming more prevalent among young adults^1,64^. Our study also shows that UPF consumption is associated with insulin resistance, a risk factor for T2D and a condition not commonly assessed in previous studies of UPFs and T2D risk. Because more than half of the total daily energy in consumption in the US is from UPFs, this modifiable risk factor is a possible target for both individual and public health interventions in preventing metabolic diseases. Future studies may incorporate additional methods of diet assessment and larger sample sizes to improve our understanding of the eating habits of young adults and the mechanisms underlying associations between UPF consumption and metabolic diseases.

## Conclusions

This prospective study found that UPF consumption is positively associated with increased risk for prediabetes among young adults. Increasing UPF consumption was also associated with impaired glucose tolerance and insulin resistance, known risk factors for the future development of T2D. This study evaluated a unique population of the youth with detailed longitudinal measurements of diet and glucose homeostasis. These findings indicate that limiting the consumption of UPFs may be an important strategy for T2D prevention among young adults.

## Figures and Tables

**Table 1:** Descriptive statistics for participant characteristics at baseline and follow-up visits.

	Baseline	Follow-Up	
			p-value^1^
UPF%, mean (SD)	20.40 (12.68)	23.60 (17.73)	0.02
Age (years), mean (SD)	19.97 (1.20)	24.07 (0.75)	-
Sex, n (%)			
Female	43 (50.59)	-	-
Male	42 (49.41)		
Ethnicity			
White	30 (35.29)	-	-
Hispanic/Latino	49 (57.65)		
Other	6 (7.06)		
BMI Category			
Normal weight	13 (15.29)	11 (12.94)	0.47
Overweight	35 (41.18)	32 (37.65)	
Obesity	37 (43.53)	42 (49.41)	
Physical Activity			
Low	-	16 (18.82)	_-_
Medium		21 (24.71)	
High		47 (55.29)	
N/A		1 (1.18)	
Type 2 Diabetes			
No Diabetes	61 (71.76)	53 (62.35)	0.17
Prediabetes/T2D	24 (28.24)	32 (37.65)	
IFG			
Normal	80 (94.12)	68 (80.00)	0.003
Abnormal	5 (5.88)	16 (18.82)	
Missing	0	1 (1.18)	
IGT			
Normal	69 (81.18)	66 (77.64)	0.82
Abnormal	16 (18.82)	15 (17.65)	
Missing	0	4 (4.71)	

1 p-values were calculated using a paired t-test for UPF% and McNemar’s test for BMI category, Type 2 Diabetes, IFG and IGT.

* Abbreviations: UPF%: percent of the diet from ultra-processed foods; BMI: body mass index; T2D: type 2 diabetes; IFG: impaired fasting glucose; IGT: impaired glucose tolerance; SD: standard deviation

**Table 2:** Descriptive statistics for glucose, insulin, and body composition outcomes at baseline and follow-up visits.

	Mean (SD)		p-value^1^
	Baseline	Follow-Up	
Glucose Measurements			
HbA1c	5.22 (0.28)	5.26 (0.52)	0.35
Fasting Glucose (mg/dL)	90.41 (7.55)	95.32 (16.61)	0.003
Two-Hour Glucose (mg/dL)	119 (26.43)	121.3 (35.04)	0.39
Glucose AUC	263.1 (44.98)	270.5 (45.11)	0.02
Insulin Measurements			
Fasting Insulin (μIU/mL)	7.44 (4.92)	13.27 (11.18)	<0.001
Two-Hour Insulin (μIU/mL)	57.19 (49.56)	88.33 (128.47)	0.03
Insulin AUC	152.74 (104.16)	186.5 (176.91)	0.20
HOMA-\varvec*β*	100.01 (63.22)	151.43 (120.26)	0.004
HOMA-IR	1.68 (1.18)	3.36 (3.73)	0.001
Matsuda Index	5.32 (3.85)	4.32 (2.83)	0.03
Body Composition			
BMI (kg/m^2^)	30.09 (4.95)	31.85 (7.03)	<0.001
Body Fat (%)	35.24 (8.13)	38.3 (8.37)	<0.001
Android/Gynoid Ratio	0.99 (0.14)	1.01 (0.15)	0.29
Fat Mass/Height^2^	10.62 (3.66)	12.21 (4.79)	<0.001
VAT mass (g)	506.4 (197.78)	594.3 (301.43)	<0.001

1 p-values were calculated using paired t-tests.

* Abbreviations: SD: standard deviation; BMI: body mass index; VAT: visceral adipose tissue; HbA1c:Hemoglobin A1c; AUC: Area Under the Curve; HOMA-*β*: homeostatic model assessment of *β*-cell function; HOMA-IR: homeostatic model assessment for insulin resistance.

**Table 3:** Odds ratios for the effect of change in UPF consumption and baseline UPF consumption on prediabetes/type 2 diabetes, impaired fasting glucose, and impaired glucose tolerance.

	OR (95% CI)	
	UPF%*Δ*^1^	UPF% at Baseline^2^
Prediabetes/T2D	1.64 (1.15, 2.50)	1.34 (0.87, 2.12)
IFG	1.22 (0.84, 1.78)	1.22 (0.71, 2.05)
IGT	2.56 (1.42, 5.86)	1.30 (0.65, 2.66)

* All outcomes were measured at the follow-up visit. Effects are scaled by 10 units.

1 Models were adjusted for age, sex, ethnicity, exercise, and UPF% at baseline.

2 Models were adjusted for age, sex, ethnicity, and UPF%Δ.

** Abbreviations: UPF%: percent of diet from ultra-processed foods; UPF%Δ: change in percent of diet from ultra-processed foods between visits; T2D: type 2 diabetes; IFG: impaired fasting glucose; IGT: impaired glucose tolerance.

**Table 4:** Effect estimates for UPF percentage change and baseline UPF consumption on body composition and glucose, insulin, and body composition measurements.

	\varvec*β* (95% CI)	
	UPF%*Δ*^1^	UPF% at Baseline^2^
Glucose Measurements		
HbA1c	0.04 (−0.04, 0.12)	−0.01 (−0.11, 0.10)
Fasting Glucose	1.77 (−0.78, 4.32)	0.47 (−2.78, 3.73)
Glucose After 120 minutes	5.72 (0.43, 11.01)	0.83 (−5.87, 7.53)
Glucose AUC	4.89 (−1.90, 11.68)	3.36 (−5.25, 11.96)
Insulin Measurements		
Fasting Insulin	0.63 (−0.97, 0.22)	2.09 (0.06, 4.12)
Insulin After 120 mins	5.36 (−12.46, 23.18)	44.75 (22.26, 67.25)
Insulin AUC	2.96 (−19.49, 25.42)	63.19 (34.84, 91.54)
HOMA-\varvec*β*	0.17 (−16.40, 16.74)	19.14 (−2.01, 40.28)
HOMA-IR	0.29 (−0.26, 0.84)	0.44 (−0.27, 1.14)
Matsuda Index	−0.38 (−0.79, 0.02)	−0.62 (−1.11, −0.13)
Body Composition		
BMI	0.41 (−0.66, 1.48)	0.82 (−0.54, 2.19)
Body Fat Percentage	0.58 (−0.33, 1.49)	1.07 (−0.07, 2.21)
Android/Gynoid Ratio	0.01 (−0.02, 0.03)	0.01 (−0.02, 0.03)
Fat Mass/Height^2^	0.17 (−0.52, 0.86)	0.47 (−0.39, 1.34)
VAT Mass	0.13 (−4.77, 4.80)	25.26 (−34.65, 85.16)

* All outcomes were measured at the follow-up visit. Effects are scaled by 10 units.

1 Models were adjusted for age, sex, ethnicity, exercise, and UPF% at baseline.

2 Models were adjusted for age, sex, ethnicity, and UPF%Δ.

** Abbreviations: UPF%: percent of diet from ultra-processed foods; UPF%Δ: change in percent of diet from ultra-processed foods between visits; BMI: body mass index; VAT: visceral adipose tissue; HbA1c: Hemoglobin A1c; AUC: Area Under the Curve; HOMA-*β*: homeostatic model assessment of *β*-cell function; HOMA-IR: homeostatic model assessment for insulin resistance.

## Data Availability

Data described in the manuscript, code book, and analytic code will be made available upon request. The data are not publicly available to protect participants’ identifiable information.
